# Efficacy of adoptive transfer of expanded fetal liver-derived precursor syngeneic and allogeneic murine NK cells against solid tumors

**DOI:** 10.1186/2051-1426-3-S2-P30

**Published:** 2015-11-04

**Authors:** Xiaoyan Liang, Wenqian Wang, Guanqiao Li, Xiaokui Zhang, Vladimir Jankovic, Uri Herzberg, Wolfgang Hofgartner, Michael T Lotze

**Affiliations:** 1 University of Pittsburgh, Pittsburgh, PA, USA; 2 University of Pittsburgh, Tsinghua University, Pittsburgh, PA, USA; 3 Celgene Cellular Therapeutics, Warren, NJ, USA

## Introduction

Clinical studies have demonstrated that adoptive transfer of allogeneic Natural Killer (NK) cells can play a therapeutic role in hematological malignancies and potentially solid tumors. We established and characterized *ex vivo* methods to obtain murine NK1.1^+^ cells from mouse fetal liver. These cells represent an orthologous cell type for expanded cells from human cord or placenta. Persistence, engraftment, expansion and anti-tumor efficacy of these fetal liver derived intermediate NK(FLiNK) cells were evaluated in allogeneic or syngeneic recipient mice after adoptive transfer. This study supports the possibility of clinical therapeutic application of *ex vivo* generated NK cells from HSC sources.

## Material and Methods

HSC were isolated from C57BL/6(B6) fetuses (day 18-20 of gestation) using a Hematopoietic Stem Cell isolation kit and cultured with a cytokine cocktail in three stages over 21 days. FLiNK cells were harvested and characterized prior to administration. MC38-luciferase^+^ cells (2X10^5^) or Renca-luciferase^+^ cells were injected into recipient mice via tail vein to establish pulmonary metastasis. Tumor bearing mice received 2.5Gy irradiation followed by 5-10X10^6^ NK cells by tail vein injection and administration of rIL-2 (200,000IU/mouse, intra-peritoneal injection twice/day for 5days). Tumor growth was measured by IVIS, lung weight, total body weight obtained, and tumor number recorded.

## Results

c-kit^+^CD16^-^NK1.1^-^CD34^-^ NK progenitor cells were successfully isolated from mouse fetal livers, and propagated over 21 days to give rise to FLiNK cells, which are CD3^-^NK1.1^+^NKp46^+^CD94^+^NKG2D^+^KLRG1^+^CD244^+^. Adoptive transfer of syngeneic FLiNK cell significantly inhibits development of mouse lung metastasis, while allogenic FLiNK showed slightly inhibitory effects. Compared with conventional splenic NK cells, FLiNK cells exhibited higher *in vitro* cytolytic activity against MC38(FLiNK 30.2±4.0 vs splenic NK 22.4±0.5, P6 B6 FLiNK cells inhibited syngeneic MC38 tumor growth in B6 mice. Survival times were significantly prolonged from the 36 days in saline (no NK) group (n=17) to 50 days in FLiNK recipients(n=15), (p < 0.05). Using allogeneic Balb/c Renca tumor bearing mice, B6 FLiNK also demonstrated anti-tumor effects, with control (no NK) survival of 25 days(n=5) compared with 30 days in the FLiNK group (n=5).

## Conclusions

This study successfully established an *ex vivo* protocol to obtain NK1.1^+^ cells from c-kit^+^CD16^-^NK1.1^-^CD34^-^NK progenitor cells from the mouse fetal liver. These FLiNK cells, on adoptive transfer, inhibit development of syngeneic and allogenic mouse lung metastasis. Proposed clinical trials of allogeneic NK cells in patients with pulmonary metastatic renal cancer are in preparation.

